# Supplementary material for: Metabolic reconstruction reveals ATP salvage as a key response to trimethoprim treatment
Source: iScience. 2026 Mar 16;29(4):115377. doi: 10.1016/j.isci.2026.115377 (PMC13091032; doi:10.1016/j.isci.2026.115377)
Supplement: Document S1. Figures S1–S6 and Tables S1–S6 [file mmc1.pdf]

iScience, Volume *29*

Supplemental information

Metabolic reconstruction reveals ATP salvage as a key response to trimethoprim treatment

Cailean Carter, Dipali Singh, John Wain, and Gemma C. Langridge

Supplementary material

Methods S1: Model properties and validation

The reactions contained within the model were modularised for ease of analysis, thereby aiding focus on or exclusion of groups of reactions. Within ScrumPy, a top-level module was loaded which imports files containing the intracellular reactions and transporters (summarised in Figure S1). The intracellular reactions included the polymerisation reactions and any manually corrected reactions (termed *E. coli*), the electron transport chain (ETC), and the cytosolic reactions generated during the construction of the model from the *E. coli* UTI89 BioCyc database v22.0. Transporter reactions encompassed the importation of M9 minimal media and atmospheric gases, and biomass export reactions that represented cellular components *E. coli* would synthesis when growing.

Fatty acid, peptidoglycan, and lipopolysaccharide synthesis pathways were condensed into a single reaction per pathway to reduce complexity. The reaction stoichiometry for each reaction is outlined below:

Fatty acid synthesis

8 acetyl-CoA + 14 NADPH + 7 ATP + 6 H^+ + H_2O ↔ palmitate + 14 NADP + 8 CoA + 7 ADP + 7 Pi

Peptidoglycan synthesis

3 L-α-alanine + PEP + NADPH + D-glutamate + 5 ATP + meso-diaminopimelate + Und-PP + 2 UDP-GlcNAc ↔ peptidoglycan + 5 ADP + 7 Pi + UMP + UDP + NADP

Lipopolysaccharide synthesis

2 CMP-Kdo + 4 ADP-L-glycero-D-manno-heptose + 3 UDP-glucose + 2 UDP-GlcNAc + 6 palmitate + 3 ATP + UDP-galactose ↔ LPS + 7 ADP + acetate + UMP + 5 UDP + 2 CMP

Transporter reactions were added for the import of adenine, glycine, methionine, and thymidine from the media. For handling mATP, four reactions were added. One reaction converted ATP to mATP at no cost and one reaction transported mATP outside the system. The other two reactions were the same as described but in reverse to track import and export of mATP (Table S2). These reactions were only included in the analysis when specified.

Some corrections were made during the validation process. Firstly, the model incorrectly contained the reaction "AIRCARBOXY-RXN" which added a carbon unit from carbon dioxide to the purine ring. In *E. coli*, this reaction was performed by two separate enzymes and used bicarbonate as the carbon donor for the purine ring according to the *E. coli* UTI89 EcoCyc v27.1 database. Reactions "RXN0-742" and "RXN0-743" which perform this step were already present in the model, thus, removing "AIRCARBOXY-RXN" from the model was the only action taken to address this. The sulphite dehydrogenase reaction was changed from reversible to forward direction to conserve internal energy balance and match the MetaCyc description of the reaction (accessed 20/06/2023). Two reactions were added to the ETC module to match the *E. coli* UTI89 EcoCyc v27.1 database:

1.10.2.2-RXN

Ubiquinols + 2 Cytochromes-C-Oxidized + 2 H^+ → 4 x_H^+ + Ubiquinones + 2 Cytochromes-C-Reduced

RXN0-5268

O_2 + 2 Ubiquinols + 8 H^+ → 8 x_H^+ + 2 H_2O + 2 Ubiquinones

The metabolic model encompassed 1265 reactions for the conversion of 1216 metabolites. However, 44.6% of reactions were unable to carry flux under steady state (namely dead reactions). Similarly, 45.1% of metabolites could not be balanced under steady state (namely orphan metabolites), these could only be produced or consumed. Nonetheless, a significant portion of dead reactions and orphan metabolites was expected due to the incomplete information available for any given bacterial organism. Similarly, the automated process of building a GSM from whole genomes included the errors and inconsistencies present in these databases and needed to be manually curated. Several aspects of the model were checked for consistency and validation (Table S3):

- Atomic balance: checked the proportion of atoms (C, N, P, S, O, and H) in substrates matched that in products for each reaction.
- Energy and redox conservation: checked ATP, NADH and NADPH were not produced in the absence of flux into the network.
- Mass conservation: similarly, checked the model was not producing compounds from nothing.
- Stoichiometry inconsistencies: checked reactions were balanced. For example, if two reactions with the same reactants and products were producing different quantities of products from the same quantity of reactants.
- Electron transport chain: checked the model produced energy from compounds in the media as expected.
- Biomass production: checked the model could synthesise all necessary biomass components.

Following these steps, the model could successfully synthesise all biomass components (Figure S2).

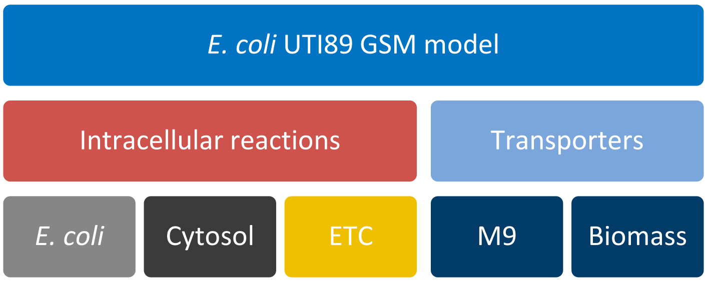

Figure S1. Modular structure of *E. coli* UTI89 GSM model.

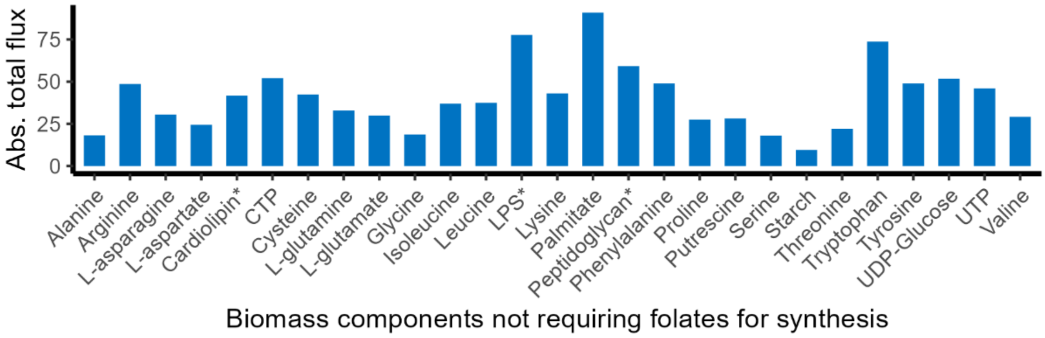

Figure S2. Biomass components not affected by trimethoprim-induced disruption of the folate pool, related to Figure 2. Bar chart of absolute total flux of LP solution to synthesise 1 unit of biomass component. Thus, asserting the model can synthesise all biomass components.

*The absolute total flux for these metabolites was divided by 10 to improve visibility of the other metabolites on the same scale.

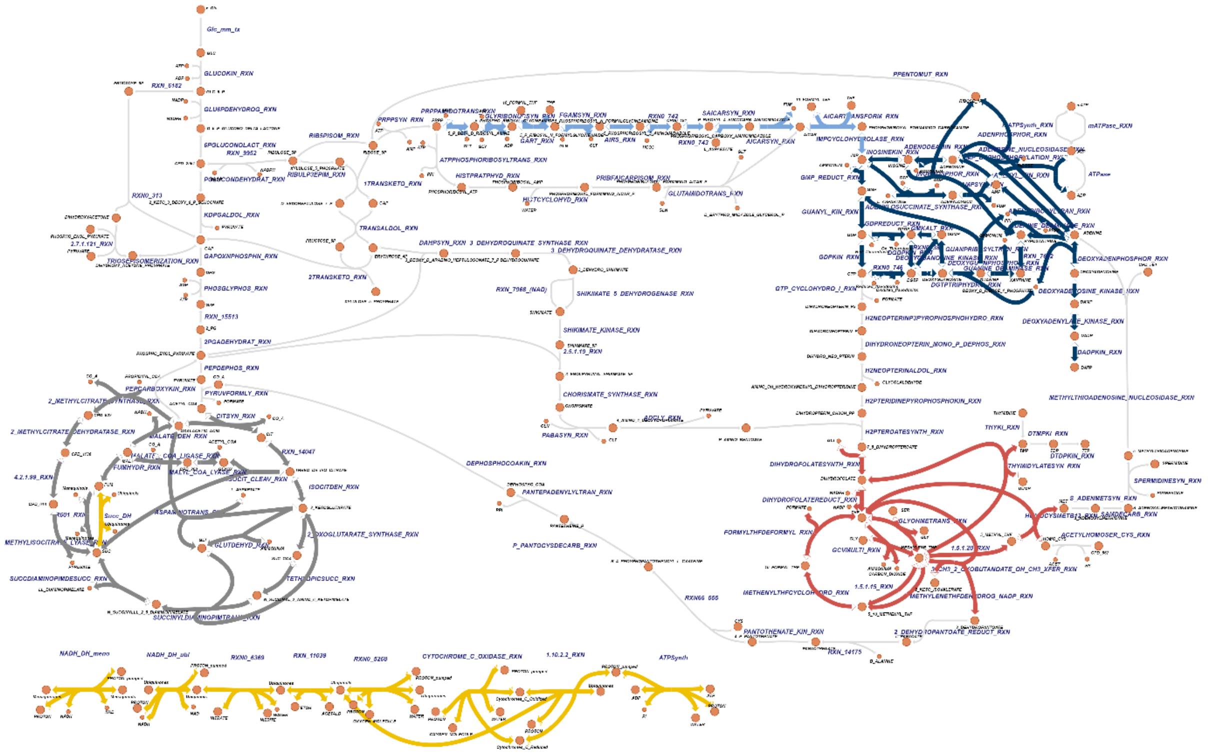

Figure S3. Network map of core metabolism in *E. coli* UTI89 GSM model, related to Figure 4. Electron transport chain (ETC) and oxidative phosphorylation (OP), tricarboxylic acid (TCA) cycle, folate associated reactions, purine biosynthesis, and purine salvage highlighted.

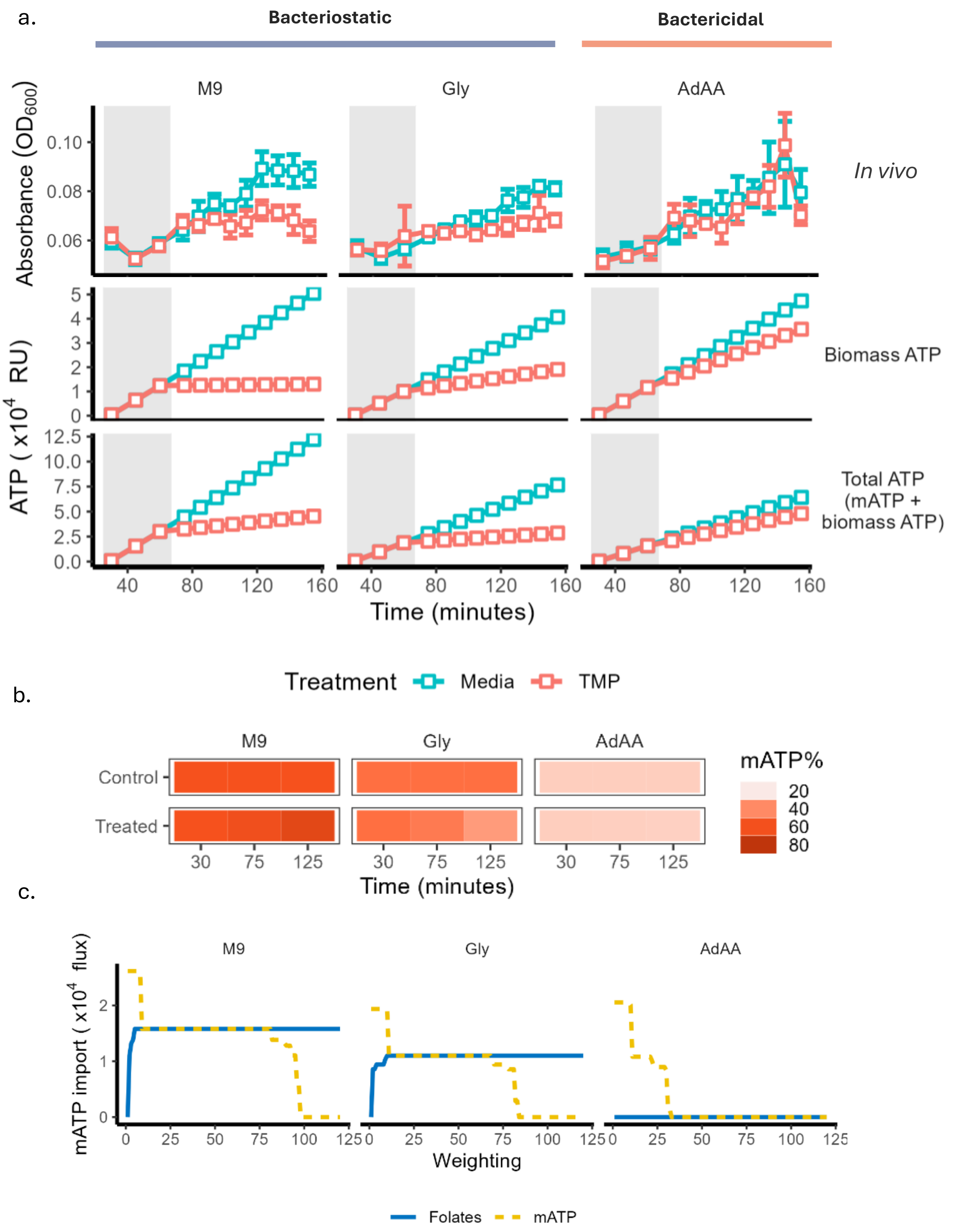

Figure S4. Preliminary incorporation of omics data, related to Figure 4. A. top) Time-course absorbance assay of a trimethoprim (TMP) susceptible clinical UPEC isolate. Assay was performed in triplicates in M9 minimal media which was either not supplemented (M9), supplemented with glycine (Gly), or with adenine, glycine, and methionine (AdAA). The grey box denotes baseline measurements before treatment of blank media or trimethoprim was added. Each data point is the mean of 4 biological replicates with error bars showing standard error mean of 95% confidence intervals. A. mid) Simulated production of biomass ATP over time. A. Bottom) Production of total ATP over time as determined by the rate of ATP synthesis from ATP bioluminescence. In both figures, the growth rate in control conditions was used for baseline time points (≤60 mins) for the treated groups. B.) Heatmap of proportion of total ATP that is maintenance ATP (mATP) from A bottom plot. C.) Change in mATP import flux as increased weighting was applied to the mATP importer. Change in flux for mATP import with increased weighting applied to folate associated reactions when optimum mATP import weighting was applied.

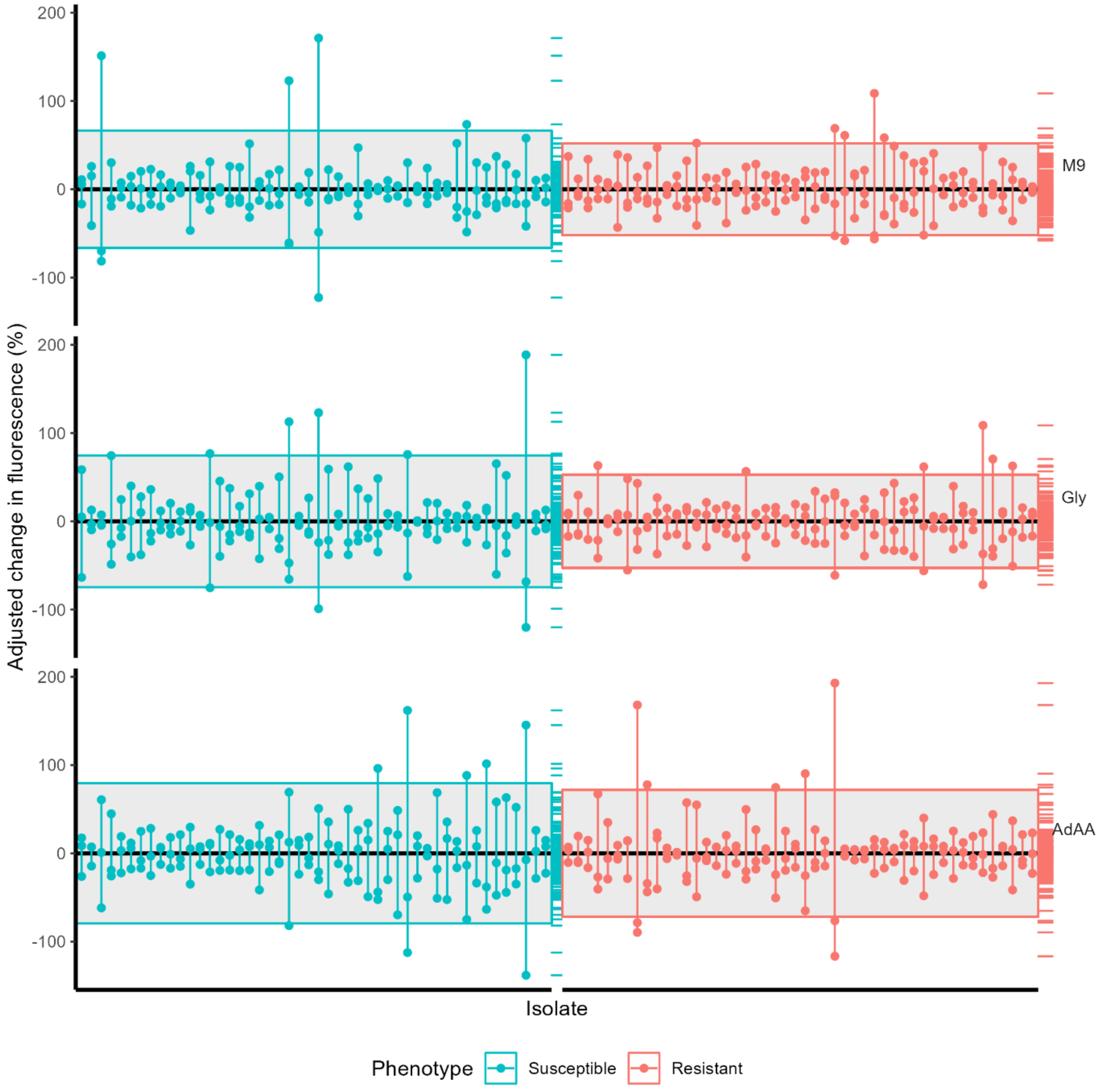

Figure S5. Distribution in change in fluorescence of three replicates per isolate, related to Figure 6. Each isolate is plotted along the x-axis with a vertical line between the three replicates. The black horizontal line indicates the population mean of zero. The rug plot on the right of each scatter plot illustrates data density across the y-axis. The grey box denotes 2 standard deviations ($\pm 2\sigma$).

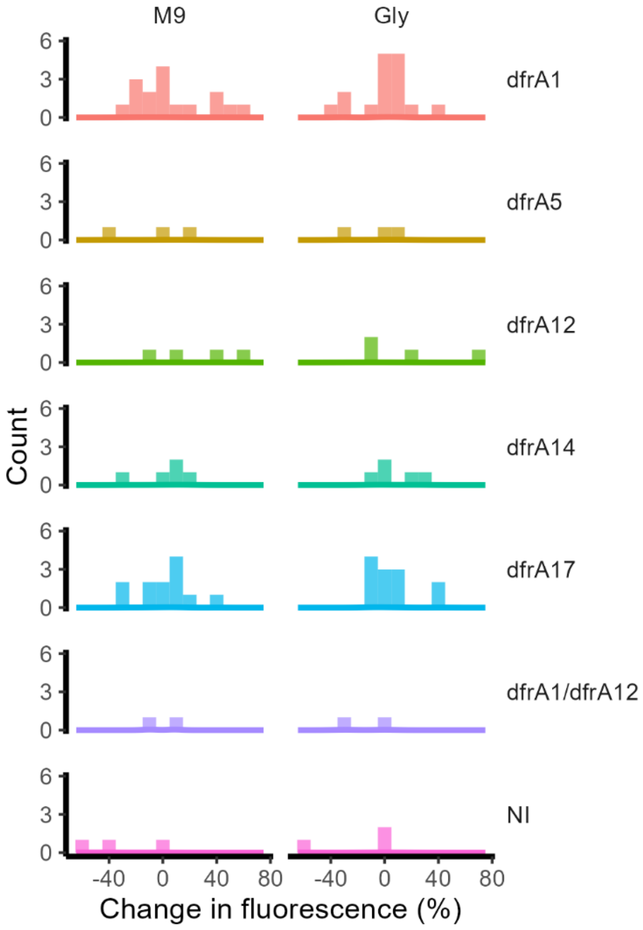

Figure S6. Histogram comparing different trimethoprim resistance mechanisms in relation to change in fluorescence in bacteriostatic conditions, related to Figure 6. NI denotes isolates where no known trimethoprim resistance determinant was identified.

Table S1. Biomass components of *E. coli* UTI89 as described in the model. All values are in mmol/gDW with default growth rate (μ) of 1.

Amino acids		RNA		Cell wall	
Alanine	0.60664	ATP	0.109351	Cardiolipin	0.003198
Arginine	0.177753	CTP	0.087659	Lipopolysaccharide	0.008367
Asparagine	0.191759	GTP	0.130251	Peptidoglycan	0.024752
Aspartate	0.191792	UTP	0.094478	Phosphatidate	0.032487
Cysteine	0.078999	**DNA**		Phosphatidylethanolamine	0.029767
Glutamine	0.188763	dATP	0.016927	Phosphatidylglycerol	0.028573
Glutamate	0.188794	dCTP	0.017002	Phosphatidylserine	0.0281
Glycine	0.862496	dGTP	0.015649	**Other**	
Histidine	0.065315	dTTP	0.015949	Putrescine	0.227814
Isoleucine	0.231768	**Cofactors**		Spermidine	0.028084
Leucine	0.360529	Acetyl-CoA	0.000214	Starch	0.138769
Lysine	0.244788	CoA	0.000135		
Methionine	0.109425	FAD	0.000176		
Phenylalanine	0.119286	5-methyl THF	0.000904		
Proline	0.200492	NAD	0.001665		
Serine	0.214286	NADH	0.000042		
Threonine	0.222253	NADP	0.000093		
Tryptophan	0.030324	NADPH	0.000279		
Tyrosine	0.080788	Succinyl-CoA	0.00007		
Valine	0.124954				

Abbreviation: gDW, gram dry weight.

Table S2. Summary of reactions involved with mATP

Reaction name	Stoichiometry
mATP_tx (importer)	x_mATP → mATP
mATPSynth-RXN	ATP → mATP
mATPase-RXN	mATP → ATP
mATP_bm_tx (exporter)	x_mATP ← mATP

Table S3. Properties of the *E. coli* UTI89 GSM model

Quantitative properties of model	Total
Reactions (excluding transporters)	1265
Transporters	77
Reactions associated with identifiable genes	1006
Metabolites	1216
Dead reactions	564
Orphan metabolites	548
Unbalanced reactions	0
Metabolites with undefined empirical formula	0
**Qualitative properties of model**	**Checked?**
Atomic balance for C, N, S, P, O, H	Yes
Energy conservation	Yes
Biomass production	Yes

Table S4. Reactions grouped into their respective pathways. Reactions are their common name or BioCyc ID when common name not available.

Pathway	Reactions
Folates	Table S5
Purine biosynthesis	Amidophosphoribosyltransferase, phosphoribosylglycinamide formyltransferase, phosphoribosylformylglycinamide cyclo-ligase, phosphoribosylaminoimidazole-succinocarboxamide synthase, phosphoribosylamine—glycine ligase, AICAR transformylase, IMP cyclohydrolase, adenylosuccinate lyase, adenylosuccinate lyase, N^5-carboxyaminoimidazole ribonucleotide mutase, 5-(carboxyamino)imidazole ribonucleotide synthase, phosphoribosylglycinamide formyltransferase 2, phosphoribosylformylglycinamide synthetase
Purine salvage	Adenine deaminase, adenosine deaminase, nucleoside phosphorylase, deoxynucleoside kinase, purine nucleoside phosphorylase, adenylate kinase, nucleoside diphosphate kinase, ribonucleoside-diphosphate reductase 1, guanylate kinase, GMP reductase, guanine deaminase, guanylate kinase, inosine/guanosine kinase, xanthine dehydrogenase, adenylosuccinate lyase, deoxyadenosine kinase, AMP phosphorylase, ribonucleoside hydrolase, adenylosuccinate synthetase, adenine phosphoribosyltransferase, guanine phosphoribosyltransferase, nucleoside triphosphate pyrophosphohydrolase, dGTP triphosphohydrolase, deoxyadenylate kinase
Tricarboxylic acid cycle	Branched-chain α-keto acid dehydrogenase complex, lipoamide dehydrogenase, branched-chain α-keto acid dehydrogenase complex, 2-oxoglutarate decarboxylase, 2-oxoglutarate synthase, aconitate hydratase A, citrate synthase, fumarase, isocitrate dehydrogenase, malate thiokinase, malate dehydrogenase, aconitate hydratase A, succinate dehydrogenase, succinate:caldariellaquinone oxidoreductase complex, dihydrolipoyltranssuccinylase, succinyl-CoA synthetase, succinate:quinone oxidoreductase, malyl-CoA lyase, malate-CoA ligase, isocitrate lyase, aspartate aminotransferase, glutamate dehydrogenase, glutamate synthase, 2-methylcitrate synthase, 2-methylcitrate dehydratase, 2-methylisocitrate lyase, fumarate reductase, succinyl-diaminopimelate desuccinylase, phosphoserine/phosphohydroxythreonine aminotransferase, tetrahydrodipicolinate succinylase,
Electron transport chain & oxidative phosphorylation	NADH:quinone oxidoreductase, NADH:menaquinone oxidoreductase, cytochrome-c-oxidase, succinate dehydrogenase, cytochrome-c-reductase, cytochrome-b-oxidase, ATP synthase, nitrate reductase (quinone), alcohol dehydrogenase (quinone)

Table S5. List of folate associated reactions. Reaction names are their common names when available, otherwise a BioCyc ID is used.

Reaction name	Stoichiometry
**AICAR transformylase**	AICAR + 10-formyl-THF ↔ FAICAR + THF
**Methylene THF dehydrogenase**	NADP + methylene-THF ↔ NADPH + 5,10-methenyl-THF
**Dehydropantoate hydroxymethyltransferase**	H_2O + 2-keto-isovalerate + methylene-THF ↔ 2-dehydropantoate + THF
**Phosphoribosylglycinamide formyltransferase**	GAR + 10-formyl-THF ↔ H^+ + FGAR + THF
**RXN-2881**	Formaldehyde + THF ↔ H_2O + methylene-THF
**Methionine synthase**	L-homocysteine + 5-methyl-THF → L-methionine + THF
**1.5.1.20-RXN**	NAD + 5-methyl-THF ← H^+ + NADH + methylene-THF
**Glycine cleavage system**	NAD + THF + glycine → CO_2 + NH_4^+ + NADH + methylene-THF
**Dihydrofolate reductase**	THF + NADP ← H^+ + NADPH + DHF
**1.5.1.15-RXN**	NAD + methylene-THF ↔ 5,10-methenyl-THF + NADH
**Thymidylate synthase**	dUMP + methylene-THF → DHF + dTMP
**Serine hydroxymethyltransferase**	L-serine + THF ↔ H_2O + glycine + methylene-THF
**Methylene THF cyclohydrolase**	5,10-methenyl-THF + H_2O ↔ H^+ + 10-formyl-THF
**Ferredoxin-dependent methylene-THF reductase**	5-methyl-THF + 2 Oxidized-ferredoxins ← 2 H^+ + 2 Reduced-ferredoxins + methylene-THF
**RXN0-1862**	UDP-4-amino-4-deoxy-L-arabinopyranose + 10-formyl-THF ↔ H^+ + UDP-4-deoxy-4-formamido-β-L-arabinopyranose + THF
**RXN-13909**	NAD + 10-formyl-THF → H^+ + 10-formyl-7,8-DHF + NADH
**5-formyl-THF cyclo-ligase**	5-formyl-THF + ATP → 5,10-methenyl-THF + Pi + ADP
**Trimethylsulfonium methyltransferase**	Trimethyl sulfonium + THF ← H^+ + 5-methyl-THF + dimethyl sulphide
**Formate—THF ligase**	Formate + ATP + THF → Pi + 10-formyl-THF + ADP
**DHF synthetase**	L-glutamate + ATP + 7,8-dihydropteroate → H^+ + DHF + Pi + ADP

Abbreviations: dUMP, deoxyuridine monophosphate; Pi, inorganic phosphate

Table S6. Luciferase ATP bioluminescence reaction stoichiometry [S1].

luciferase + ATP + D-luciferin → luciferase(luciferyl-adenylate) + PPi	(1)
luciferase(luciferyl-adenylate) + O_2 → luciferase(oxyluciferin; AMP) + CO_2	(2)
luciferase(oxyluciferin; AMP) → luciferase(oxyluciferin; AMP) + photon	(3)
luciferase(oxyluciferin; AMP) → luciferase + oxyluciferin + AMP	(4)
ATP + O_2 + D-luciferin → oxyluciferin + AMP + PPi + CO_2 + photon	(Net)

References

1. Lundin, A. (2000). Use of firefly luciferase in ATP-related assays of biomass, enzymes, and metabolites. Methods Enzymol. *305*, 346-370. 10.1016/s0076-6879(00)05499-9.
